# Rapid detection of human blood in triatomines (kissing bugs) utilizing a lateral flow immunochromatographic assay - A pilot study

**DOI:** 10.1590/0074-02760190047

**Published:** 2019-05-30

**Authors:** Norman L Beatty, Nicole Behrens-Bradley, Maria Love, Finn McCants, Shannon Smith, Justin O Schmidt, Sarah A Hamer, Patricia L Dorn, Nafees Ahmad, Stephen A Klotz

**Affiliations:** 1 University of Arizona College of Medicine, Department of Medicine, Division of Infectious Diseases, Tucson, AZ, United States of America; 2 University of Arizona College of Medicine, Department of Immunobiology, Tucson, AZ, United States of America; 3 Loyola University New Orleans, Department of Biological Sciences, New Orleans, LA, United States of America; 4 Southwestern Biological Institute, Tucson, AZ, United States of America; 5 Texas A&M University, Veterinary Medicine and Biomedical Sciences, College Station, TX, United States of America

**Keywords:** triatomine, kissing bug, human blood detection, blood sources, Chagas disease

## Abstract

**OBJECTIVES:**

We tested a rapid and specific immunochromatographic assay (that detects human blood in forensic samples) to determine if human blood was present in triatomines and their fecal excreta.

**METHODS:**

We fed *Triatoma rubida* human blood (positive control) or mouse blood (negative control) and performed the assay on the abdominal contents and fecal excreta. Triatomine field specimens collected in and around human habitations and excreta were also tested.

**FINDINGS:**

The assay was positive in triatomines fed human blood (N = 5/5) and fecal excreta from bugs known to have ingested human blood (N = 5/5). Bugs feeding on mice (N = 15/15) and their fecal excreta (N = 8/8) were negative for human blood. Human blood was detected in 47% (N = 23/49) triatomines, representing six different species, collected in the field.

**MAIN CONCLUSIONS:**

The pilot study shows that this rapid and specific test may have applications in triatomine research. Further study is needed to determine the sensitivity of this assay compared to other well-established techniques, such as DNA- and proteomics-based methodologies and the assay’s application in the field.

Triatomines are hematophagous insects belonging to the Reduviidae family. They are commonly known as “kissing bugs” in the United States and “benchuca,” “vinchuca,” or “barbeiro” in other regions of Latin America. Triatomines are vectors of *Trypanosoma cruzi*, a protozoan parasite that is the causative agent of Chagas disease.^1^ Human exposure to triatomines can occur in sylvatic, peridomestic, and domestic settings. Vector blood meal analysis is important for understanding vector ecology and assessing efficacy of Chagas disease control strategies.^2^ Several techniques have been developed using DNA- and proteomics-based methodologies in an attempt to determine the blood meal sources of triatomines.^3^ ^,^ ^4^ ^,^ ^5^ ^,^ ^6^ ^,^ ^7^ These procedures are sensitive and specific for identifying a blood meal source, but are not readily adaptable to field use. They require access to a laboratory with sophisticated equipment and trained personnel, are labor intensive, and are vulnerable to contamination and degradation of the blood meal.^3^ ^,^ ^4^ ^,^ ^5^ ^,^ ^6^ ^,^ ^7^ The Rapid Stain Identification of Human Blood (RSID™ Blood) is a lateral flow, immunochromatographic assay detecting as little as 1 µL of human blood in forensic samples within 10 min following 1-2 h of specimen preparation. We conducted a preliminary study using this assay on triatomines and their excreta fed mouse or human blood and also numerous field specimens.

## MATERIALS AND METHODS

*Ethics* - A colony of *Mus musculus* (Harlan Laboratories, Madison, WI) was used for mouse feeding experiments. All procedures using mice were approved by the Southwestern Biological Institute, Tucson, Arizona, USA Animal Care and Use Committee following international standards.^7^ Human venous blood was provided from a volunteer after informed consent.

*Control triatomines for blood testing* - Laboratory-raised fifth instar *Triatoma rubida* were used for the mouse and human blood experiments. This triatomine colony originates from a 2009 collection of wild-caught *T. rubida* from Tucson, Arizona, USA. The *T. rubida* colony is maintained as described by Keller et al.^7^ Fifteen laboratory-raised fifth instar *T. rubida* were fed mouse blood for 30-60 min or until engorgement, at which time the mouse was removed. Five laboratory-raised fifth instar *T. rubida* having only fed previously on mouse blood (approximately six weeks prior) were fed human blood through an artificial membrane feeding apparatus. Heparinized-venous blood was pipetted into a sterile plastic 4 mL collection tube, and a layer of parafilm was placed over the top. Each tube was warmed to 37ºC using a heated water bath. The insects were placed in the center of the triangular apparatus, with 12 human blood-filled tubes available for feeding. Each insect was allowed to feed for 30-60 min or until visibly engorged. Each engorged insect was removed and placed in a 15x20 cm plastic container with a lid allowing ambient air access, an absorbent sponge water source, a piece of cardboard for housing, and filter paper on the bottom of the container to collect fecal excreta. The temperature was maintained at 26ºC. Laboratory-raised *T. rubida* were sacrificed by freezing at -20ºC.

**Fig. 1:**
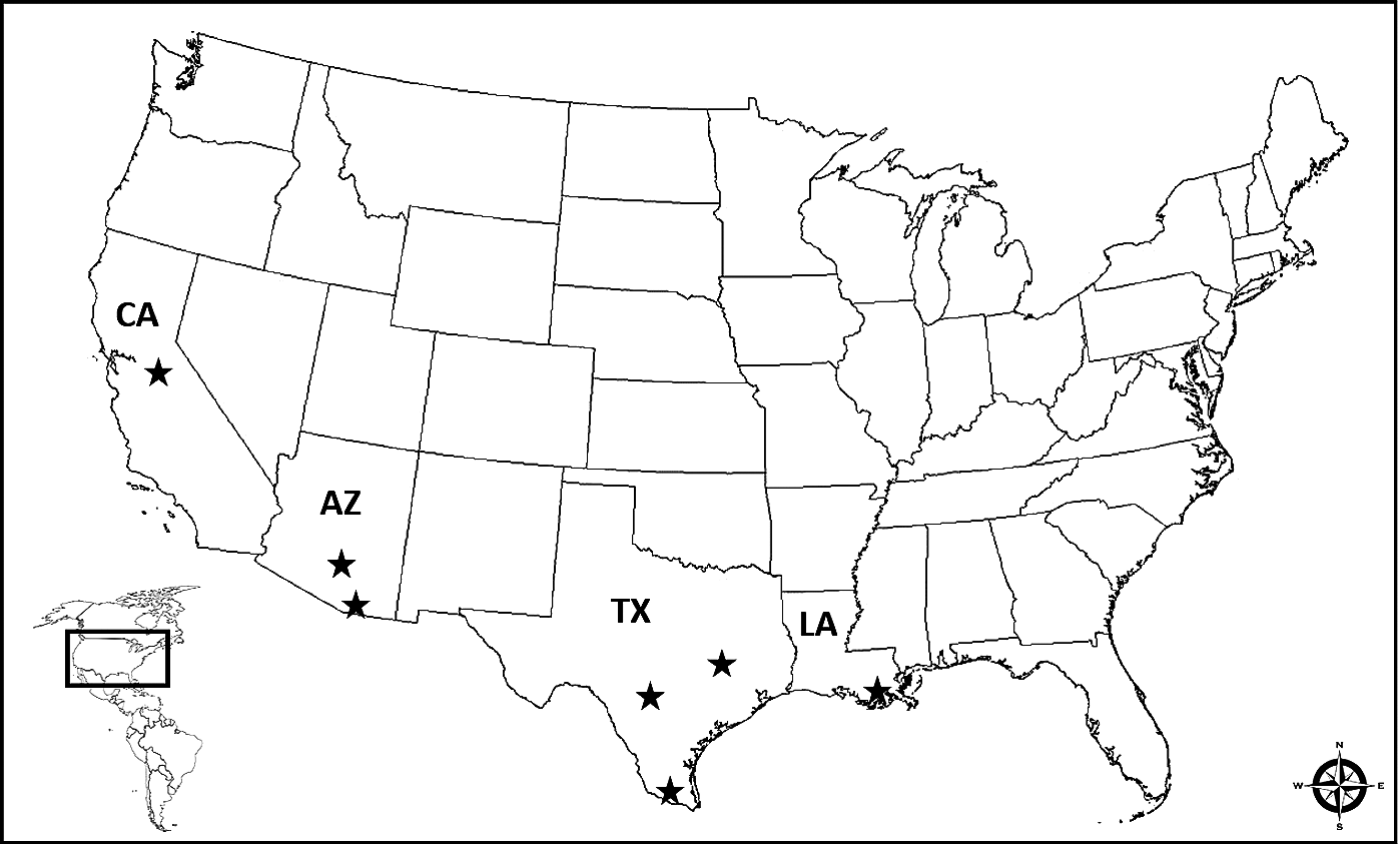
regions (black stars) where triatomine field specimens were provided from the states of Arizona (AZ), California (CA), Louisiana (LA), and Texas (TX) within the USA.

*Triatomine field specimens* - Triatomines (*T. rubida*, *T. protracta*, *T. recurva*, *T. sanguisuga*, *T. gerstaeckeri*, *T. lecticularia*) were collected using ultraviolet nighttime trapping techniques and/or brought in by homeowners from different citizen science projects from Arizona, California, Louisiana, and Texas for analysis.^8^ Triatomines were collected within a residence or close vicinity of a residence from 08/2013 to 10/2017. Specimens were provided from seven different regions from the states of Arizona, California, Louisiana, and Texas in the United States of America (Fig. 1). Each specimen was kept frozen at -20ºC, dried at room temperature, or placed in 100% ethanol for storage after collection and prior to testing.

**Fig. 2:**
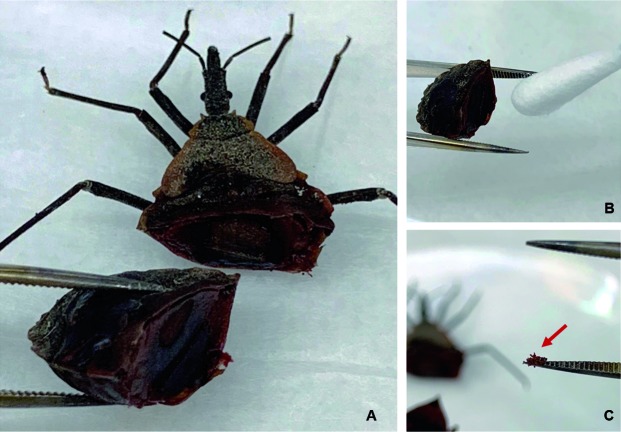
*Triatoma recurva* nymph from field collection after dissection with visible blood meal (A). A cotton-tip can be used when the blood meal is in the liquid form (B) or a dried blood meal sample can be taken (C) after dissecting an engorged triatomine.

*Preparation of triatomines for analysis* - Frozen specimens were thawed for approximately 10 min at room temperature prior to dissection. Triatomine specimens were thoroughly washed in sterile water, patted dry, and allowed to air-dry at room temperature for 2 h. The distal two-thirds of the adult and nymph abdomens were excised using a sterile razor blade on a Petri dish and examined for visible blood meal (Fig. 2A). If a triatomine from the field collection did not have a visible blood meal after dissection of the abdomen, it was not included in the study. First and second instars with visible blood meals were used whole for extraction. Triatomines from our control groups were alive until the designated time for testing and then sacrificed.

**Fig. 3:**
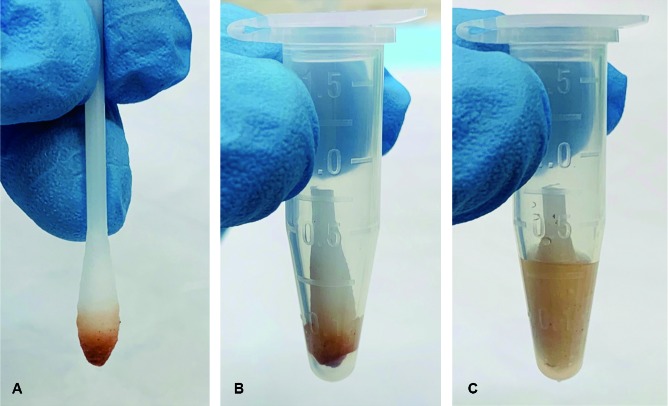
using a cotton tip applicator to collect a blood meal sample (A), the tip with the collected blood sample is cut-off and placed into an Eppendorf (B), and 200 µL of RSID™ Blood Universal Buffer is added (C).

*Blood meal extraction from triatomines* - All triatomine specimens were handled with sterile tweezers and gloved hands. A sterile cotton tip applicator was placed into visibly bloody abdominal contents of a bug to collect a sample (Fig. 2B). When blood meal samples were taken from dried triatomine specimens (coagulated blood), a single 25 mg sample was collected (Fig. 2C; red arrow) and placed into 1.5 mL Eppendorf tubes. A liquid blood meal sampled with a sterile cotton tip (Fig. 3A) was placed into the Eppendorf and the tip cut off (Fig. 3B). The manufacturer’s directions for the RSID™ Blood were followed. Briefly, RSID™ Blood Universal Buffer (200 µL) was added to each sample and the tube vortexed for 30 s. Samples were suspended in buffer for 2 h and vortexed at the 1 and 2 h marks for 30 s. Dry coagulated blood broke apart during vortexing and physical agitation with the micropipette and went into solution.

*Extraction of triatomine fecal excreta for testing* - Fecal excreta collected on filter paper from triatomines were cut out using sterile instruments (20 mm^2^) and placed into a 1.5 mL Eppendorf tube. Fecal excreta from field triatomine specimens submitted in plastic or glass containers was removed by scraping with a sterile blade. Fecal excreta was extracted for testing using the same protocol as the dried blood meal specimens.

**Fig. 4:**
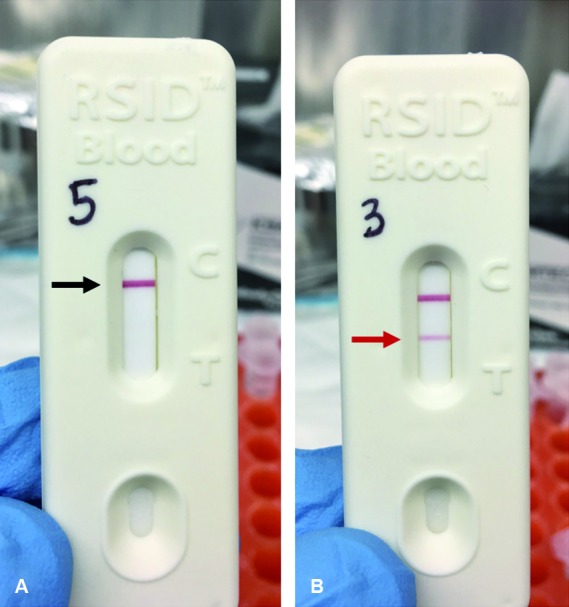
a negative result is when a single red line located at the control line (A ― black arrow) and a positive result is when a red line is located at both the control and test lines (B ― red arrow).

*Testing triatomines and fecal excreta for human* blood - A 20 µL aliquot from the sample suspended in universal buffer (Fig. 3C) was combined with 80 µL of the RSID™ Blood Running Buffer (provided in kit). The sample was gently mixed with the micropipette prior to being placed onto the cassette window. The assay was read after 10 min. A visible red line should be present at the control (C) position on each test. Lack of a visible redline at the control (C) position indicates a failed test. A single visible red line only at the control (C) position indicates a negative result and human blood was not detected (Fig. 4A; black arrow). Visible red lines at the control (C) and the test (T) positions indicated a positive result (Fig. 4B; red arrow). As recommended by the manufacturer, each test run was accompanied by a positive and negative control. The positive control group consisted of a single drop of human blood, collected on a cotton swab, and extracted using techniques described above. The negative control consisted of adding 20 µL of universal buffer with 80 µL of the running buffer. Both the positive and negative controls were run in parallel with the experimental specimens.

## RESULTS

*Triatomine controls* - Laboratory-raised *T. rubida* (N = 5/5) fed human blood tested positive with the assay after dissection after 12 h, 3, 5, 7, and 14 days post-feeding. No triatomine molted after ingesting human blood in this study and all had visible bloody contents when dissected. The laboratory-raised *T. rubida* only fed on mouse blood had visible evidence of a blood meal after dissection, and all tested negative for human blood (N = 15/15).

*Triatomine field specimens* - Among the 75 triatomines collected in the field and analyzed, 65% (N = 49/75) had a visible blood meal after dissection (N = 36 adult specimens; N = 13 nymphal specimens). Overall, the detection of human blood was found to be 47% (N = 23/49) among the field specimens with a visible blood meal (N = 9/20 (45%) Arizona specimens; N = 1/1 (100%) California specimen; N = 4/7 (57%) Louisiana specimens; N = 9/21 (42%) Texas specimens) (Table I). Between the adult and nymph life stages, 56% (N = 20/36) of the adult specimens were positive for human blood as opposed to 23% (N = 3/13) of the nymphal specimens (Table II). Two *T. rubida* and one *T. protracta* field specimens found in bed by bite victims had visible blood meals and tested positive for human blood by the assay (N = 3/3).

**TABLE I:** Field triatomines that were tested with RSID™ Blood

*Triatoma* spp.	Location(s)	Date(s) of triatomine collection (MM/YYYY)	Collection site	Positive RSID™ Blood results (blood meal)
*T. rubida*	Bisbee, AZ Tucson, AZ	08/2017 - 01/2018	I: N = 2 O: N = 0 U: N = 3	I: N = 2/2 (100%) O: n/a U: N = 2/3 (66%) N = 4/5 (80%)
*T. protracta*	Mariposa, CA Tucson, AZ	08/2017 - 01/2018	I: N = 1 O: N = 0 U: N = 1	I: N = 1/1 (100%) O: n/a U: N = 1/1 (100%) N = 2/2 (100%)
*T. recurva*	Bisbee, AZ Tucson, AZ	06/2017 - 10/2017	I: N = 8 O: N = 0 U: N = 6	I: N = 1/8 (13%) O: n/a U: N = 3/6 (50%) N = 4/14 (28%)
*T. sanguisuga*	Bayou Gauche, LA Lexington, TX	08/2013 - 08/2017	I: N = 0 O: N = 2 U: N = 7	I: n/a O: N = 2/2 (100%) U: N = 4/7 (57%) N = 6/9 (66%)
*T. gerstaeckeri*	Moore, TX Mission, TX	05/2016 - 07/2016	I: N = 1 O: N = 16 U: N = 0	I: N = 0/1 (0%) O: N = 6/16 (38%) U: n/a N = 6/17 (35%)
*T. lecticularia*	Lexington, TX	04/2016	I: N = 1 O: N = 0 U: N = 0	I: N = 1/1 (100%) O: n/a U: n/a N = 1/1 (100%)
Unknown spp.	Lexington, TX	02/2016	I: N = 1 O: N = 0 U: N = 0	I: N = 0/1 (0%) O: n/a U: n/a N = 0/1 (0%)
				I: N = 5/14 (36%) O: N = 8/18 (44%) U: N = 10/17 (59%) Total N = 23/49 (47%)

I: inside residence; O: outside residence; U: unknown.

**TABLE II:** Field triatomines life stages that were tested with RSID™ Blood

*Triatoma* spp.	Life stage	Positive RSID™ Blood results (blood meal)
*T. rubida*	Nymph (N = 0)	n/a
	Adult (N = 5)	N = 4/5 (80%)
*T. protracta*	Nymph (N = 0)	n/a
	Adult (N = 2)	N = 2/2 (100%)
*T. recurva*	Nymph (N = 12)	N = 3/12 (25%)
	Adult (N = 2)	N = 1/2 (50%)
*T. sanguisuga*	Nymph (N = 0)	n/a
	Adult (N = 9)	N = 6/9 (66%)
*T. gerstaeckeri*	Nymph (N = 0)	n/a
	Adult (N = 17)	N = 6/17 (35%)
*T. lecticularia*	Nymph (N = 0)	n/a
	Adult (N = 1)	N = 1/1 (100%)
Unknown spp.	Nymph (N = 1)	N = 0/1 (0%)
	Adult (N = 0)	n/a

*Triatomine fecal excreta* - Eight different laboratory-raised *T. rubida* that fed only on mouse blood provided eight fecal excreta samples; all tested negative (N = 8/8) using the assay. Three triatomines that tested positive from our field collection (two of which were known to have fed on a human) provided three different fecal excreta samples for testing, all tested positive (N = 3/3). In addition, laboratory-raised *T. rubida* that were fed human blood provided two different fecal excreta samples, both were positive (N = 2/2) using the assay.

## DISCUSSION

Determining whether a triatomine has fed on humans can be challenging. Although DNA- and proteomics-based methods are powerful techniques to analyze triatomine blood meal sources, they are not without difficulties. DNA-based methods that are used for these investigations typically require high-quality DNA from a recently fed insect vector.^9^ ^,^ ^10^ ^,^ ^11^ ^,^ ^12^ The success of determining a blood host in insects with high levels of engorgement (like those used in the current study) is markedly higher than in insects with low or non-detectable engorgement when using DNA-based polymerase chain reaction (PCR) amplification techniques.^13^ However, false-positive detection of human DNA not acquired from a blood meal is an important limitation of this detection methodology.^5^ A study conducted by Keller et al.^7^ compared the effectiveness of short interspersed nuclear elements (SINE)-based PCR and liquid chromatography tandem mass spectrometry (LC-MS/MS) in laboratory-raised *T. protracta* in detection of laboratory mouse (*Mus musculus*) hemoglobin and albumin from a blood meal. LC-MS/MS was able to detect mouse hemoglobin up to four weeks post-feeding and 12 weeks post-molting in *T. protracta* whereas, the DNA-based detection technique using mouse-specific SINE-DNA was able to detect mouse blood up to one-week post-feeding, and did not detect mouse blood in the post-molt triatomines.^7^ Various proteomics-based techniques utilizing mass spectrometry detected specific peptides accurately for a wide variety of host blood sources. This technology has several draw backs, including need of a facility with a proteomics platform and trained personnel, increased expense compared to other methodologies, species-species peptide mismatching, blood meal protein degradation, and a need for a blood meal reference spectra in the database.^7^ ^,^ ^9^ ^,^ ^10^ ^,^ ^12^ ^,^ ^14^ ^,^ ^15^ Serologic tools to detect a blood meal source have been reported and include precipitin and antibody tests. Although these tests are easy to perform, they require specific antibodies from potential host species unique to the geographic region of study. Cross-reactivity with these serologic investigations was demonstrated between various host species, leading to suboptimal species-specific accuracy.^16^ ^,^ ^17^ ^,^ ^18^ ^,^ ^19^

The RSID™ Blood is a lateral flow assay utilizing both capture and detection antibodies specific against a human sialoglycoprotein found on the cell surface of erythrocytes known as glycophorin A (GYPA). Glycophorin A is a transmembrane sialoglycoprotein that is an essential component of the erythrocyte cell membrane. It is responsible for the majority of the membrane’s overall negative charge.^20^ Among the major proteins of the human erythrocyte cell membrane, GYPA is estimated to be found in high abundance at 1000 monomers per cell x 10^3^. Band 3 which binds to GYPA is the only other membrane protein in higher abundance.^20^ Validation studies conducted by the manufacturer showed this assay to be highly specific and negative when blood from other animals, including: dog, ferret, skunk, cat, cow, pig, chicken, horse, goat, opossum, owl, turtle, elk, deer, tiger, alpaca, and non-human primates [orangutan, gorilla, spider monkey, pygmy chimpanzee (bonobo), and baboon] was tested.^21^ The RSID™ Blood assay was previously used by us to determine if argasid ticks (*Argas cooleyi*) and swallow bugs (*Oeciacus vicarious*) were feeding on humans during a hospital infestation associated with nesting cliff swallows on the building’s exterior. Human blood was detected in 17% of the argasid ticks and none of the swallow bugs.^22^

Triatominae are hematophagous insects of the order of Hemiptera, having evolved to feed on vertebrate blood. Hematophagy among triatomines requires the ingestion of high amounts of blood proteins during a feed. The digestive physiology of this blood meal is not entirely understood but multiple midgut digestive lysosomal-like cathepsins, such as Cathepsin D-like aspartic-proteinases, Cathepsin-L like cysteine-proteinases, as well as aminopeptidases and carboxypeptidases have been implicated in the digestion of these blood proteins.^23^ ^,^ ^24^ Recently an in depth analysis has revealed that the *Rhodnius prolixus* genome contains 433 protease coding genes, belonging to 71 protease families. Seven peptidase families were found to be in higher gene numbers when compared to other arthropod genomes; with a further analysis indicating that the protease family A1 might have participated in the triatominae adaptation to hematophagy.^24^ Human sialoglycoproteins, such as GYPA, should be digested by these triatominae digestive peptidases and proteinases, but the exact physiology behind this digestion has not been described. The epitope(s) used in this assay are not public and are proprietary, but may involve a region of this sialoglycoprotein that remains intact for a period of the digestion or throughout the entire process. More study is need to better understand the dynamics of human sialoglycoprotein digestion within the triatomine digestive tract.

The rapid lateral flow immunochromatographic assay was able to detect a human blood meal up to at least two weeks post-feeding based on the experimentally fed insects. Furthermore, 47% of our triatomine field specimens had evidence of human blood. This triatomine collection was considered peridomestic, given that the bugs were collected in and around human dwellings. Greater than two-thirds of the field collection came from inside or outside a home indicating exposure to human beings. Some of the bug specimens were captured after biting and all of these were positive by assay. All bugs not fed human blood were negative by this assay. Tests of triatomine fecal excreta using this assay were successful at detecting human blood. It is unclear why human GYPA was detectable in fecal excreta samples but it may be due to undigested GYPA or fragments of this sialoglycoprotein. Undigested blood components are found in hematophagous insects, such as triatomines, and leads to the dark brown or black appearance of the dejecta (due in part to hemoglobin).^25^ Detection of the heme component of hemoglobin using the phenolphthalein test in the dejecta of hematophagous insects and arthropods was used to show infestation of human dwellings.^26^ ^,^ ^27^ ^,^ ^28^ The limitation of the phenolphthalein test is that it is not specific to human blood, but reacts with heme, which is found in the majority of cold- and warm-blooded vertebrates.^25^ In our experiments, the RSID™ Blood was able to successfully detect human blood in fecal excreta of triatomines and may be useful when evaluating suspected dejecta of triatomines and other hematophagous insects.

This test may be useful in triatomine research and possibly fieldwork when experiments need to be carried out in a field setting. Potential examples of its use include determining if triatomines are taking blood meals from humans in low-resource or rural regions where triatomines are endemic. Another use would be in homes where triatomines are discovered. This has become a problem in the United States, where individuals think they are fed upon by triatomine bugs.^29^ ^,^ ^30^ This assay was able to successfully detect human blood in triatomines preserved in ethanol, kept frozen, or dried from triatomine specimens collected years before. Utilizing this simple assay to determine if a triatomine fed on humans or assaying triatomine fecal excreta for human blood, could help quantify triatomine/human contact and estimate the risk of Chagas transmission within a home, village, or community. More study is needed to define field readiness for this assay. Field experiments are needed in order to develop such a protocol.

Since this is a pilot study to assess feasibility and practicality of utilizing a forensic assay in a new and innovative way, there are several limitations to discuss. It is unclear whether these methods can be applied to the field until studied in a field setting. Also, our main objective was to assess whether this assay would indeed detect human blood found in a triatomine with a visible blood meal. The sample size was small but sufficient to show that this assay is accurate. We did test the assay on laboratory-raised *T. rubida* that were only fed mouse blood (which were all negative), but we did not test the field triatomine specimens that did not have a visible blood meal. Furthermore, we need to evaluate the degree of triatomine engorgement, blood meal intake quantities, differences in specimen preservation (ethanol, freezing, dried) and post-molting insects after known human blood meal to assess sensitivity in regards to these perimeters. A more robust study is needed and being planned to evaluate these questions and compare this assay to other methods such DNA- and proteomic based techniques. Several limitations of the RSID™ Blood technique exist. Although many genera of animals’ blood were tested, it is unknown whether the assay will have cross-reactivity with animal blood not included in the validation panel.^21^ During our testing the RSID™ Reader was not available. It is a device designed to perform an optical interrogation of the test cassette and is useful in resolving ambiguous (very faint band) results, but is not required for correct or proper interpretation of strip test results.^31^ In our study we did not experience any ambiguous results. Another limitation is that we only tested triatomines with a visible blood meal. More investigation is needed to assess if human blood can be detected in triatomines without gross evidence of blood after dissection. Additionally, while this assay can provide quick information specifically about insect feeding on humans, various research, vector control, and veterinary initiatives may benefit from knowledge of vector feeding on non-human hosts for which other methods of blood meal analysis must be used. Among our triatomine field specimens, we detected human blood in 47% of the collection. This may not represent a random selection given these specimens were provided from circumstances of exposure to human dwellings, and sometime with reported known triatomine bite.

## References

[1] Bern C, Kjos S, Yabsley MJ, Montgomery SP (2011). Trypanosoma cruzi and Chagas' disease in the United States. Clin Microbil Rev.

[2] Pellecer MJ, Dorn PL, Bustamante DM, Rodas A, Monroy MC (2013). Vector blood meals are an early indicator of the effectiveness of the Ecohealth approach in halting Chagas transmission in Guatemala. Am J Trop Med Hyg.

[3] Lucero DE, Ribera W, Pizarro JC, Plaza C, Gordon LW, Pena R (2014). Sources of blood meals of sylvatic Triatoma guasayana near Zurima, Bolivia, assayed with qPCR and 12S cloning. PLoS Negl Trop Dis.

[4] Waleckx E, Suarez J, Richards B, Dorn PL (2014). Triatoma sanguisuga blood meals and potential for Chagas disease, Louisiana, USA. Emerg Infect Dis.

[5] Keller JI, Ballif BA, St (2017). Clair RM, Vincent JJ, Monroy MC, Stevens L Chagas disease vector blood meal sources identified by protein mass spectrometry. PLoS One.

[6] Orantes LC, Monroy C, Dorn PL, Stevens L, Rizzo DM, Morrissey L (2018). Uncovering vector, parasite, blood meal and microbiome patterns from mixed-DNA specimens of the Chagas disease vector Triatoma dimidiata. PLoS Negl Trop Dis.

[7] Keller JI, Schmidt JO, Schmoker AM, Ballif BA, Stevens L (2018). Protein mass spectrometry extends temporal blood meal detection over polymerase chain reaction in mouse-fed Chagas disease vectors. Mem Inst Oswaldo Cruz.

[8] Curtis-Robles R, Wozniak EJ, Auckland LD, Hamer GL, Hamer SA (2015). Combining public health education and disease ecology research using citizen science to assess Chagas disease rntomological risk in Texas. PLoS Negl Trop Dis.

[9] Niare S, Berenger J-M, Dieme C, Doumbo O, Raoult D, Parola P (2016). Identification of blood meal sources in the main African malaria mosquito vector by MALDI-TOF MS. Malar J.

[10] Ӧnder Ӧ, Shao W, Kemps BD, Lam H, Brisson D (2013). Identifying sources of tick blood meals using unidentified tandem mass spectral libraries. Nat Commun.

[11] Ngo KA, Kramer LD (2003). Identification of mosquito bloodmeals using polymerase chain reaction (PCR) with order-specific primers. J Med Entomol.

[12] Tandina F, Laroche M, Davoust B, Doumbo OK, Parola P (2018). Blood meal identification in the cryptic species Anopheles gambiae and Anopheles coluzzii using MALDI-TOF MS. Parasite.

[13] Curtis-Robles R, Meyers AC, Auckland LD, Zecca IB, Skiles R, Hamer SA (2018). Parasitic interactions among Trypanosoma cruzi, triatomine vectors, domestic animals, and wildlife in Big Bend National Park along the Texas-Mexico border. Acta Trop.

[14] Laskay UA, Breci L, Vilcins IM, Dietrich G, Barbour AG, Piesman J (2013). Survival of host blood proteins in Ixodes scapularis (Acari Ixodidae) ticks: a time course study. J Med Entomol.

[15] Laskay UA, Burg J, Kaleta EJ, Vilcins IM, Telford Iii SR, Barbour AG (2012). Development of a host blood meal database de novo sequencing of hemoglobin from nine small mammals using mass spectrometry. Biol Chem.

[16] Souza RCM, Soares AC, Alves CL, Lorosa ES, Pereira MH, Diotaiuti L (2011). Feeding behavior of Triatoma vitticeps (Reduviidae Triatominae) in the state of Minas Gerais, Brazil. Mem Inst Oswaldo Cruz.

[17] Fyodorova MV, Savage HM, Lopatina JV, Bulgakova TA, Ivanitsky AV, Platonova OV (2006). Evaluation of potential West Nile virus vectors in Volgograd region, Russia, 2003 (Diptera Culicidae): species composition, bloodmeal host utilization, and virus infection rates of mosquitoes. J Med Entomol.

[18] Gomes B, Sousa CA, Vicente JL, Pinho L, Calderon I, Arez E (2013). Feeding patterns of molestus and pipiens forms of Culex pipiens (Diptera Culicidae) in a region of high hybridization. Parasit Vectors.

[19] Canals M, Cruzat L, Molina MC, Ferreira A, Cattan PE (2001). Blood host sources of Mepraia spinolai (Heteroptera Reduviidae), wild vector of Chagas disease in Chile. J Med Entomol.

[20] Lux IV SE (2016). Anatomy of the red cell membrane skeleton unanswered questions. Blood.

[21] RSID-Blood RSID(tm).

[22] Beatty NL, Klotz SA, Elliott SP (2017). Hematophagous ectoparasites of cliff swallows invade a hospital and feed on humans. Clin Infect Dis.

[23] Garcia ES, Genta FA, de Azambuja P, Schaub GA (2010). Interactions between instestinal compounds of triatomines and Trypansoma cruzi. Trends Parasitol.

[24] Henriques BS, Gomes B, Costa SG, Moraes CS, Mesquita RD, Dillon VM (2017). Genome wide mapping of peptidases in Rhodnius prolixus identification of protease gene duplications, horizontally transferred proteases and analysis of peptidase A1 structures, with considerations on their role in the evolution of hematophagy in triatominae. Front Physiol.

[25] Wigglesworth VB (1931). The physiology of excretion in a blood-sucking insect, Rhodnius prolixus (Hemiptera, Reduviidae) I. Composition of the urine. J Exp Biol.

[26] Gürtler RE, Oneto ML, Cecere MC, Castañera MB, Canale DM (2001). A simple method to identify triatomine (Hemiptera Reduviidae) feces in sensing devices used in vector surveillance programs. J Med Entomol.

[27] Vazquez-Prokopec GM, Ceballos LA, Salomón OD, Gürtler RE (2002). Field trials of an improved cost-effective device for detecting peridomestic populations of Triatoma infestans (Hemiptera Reduviidae) in rural Argentina. Mem Inst Oswaldo Cruz.

[28] Cecere MC, Vázquez-Prokopec GM, Ceballos LA, Gurevitz JM, Zárate JE, Zaidenberg M (2006). Comparative trial of effectiveness of pyrethroid insecticides against peridomestic populations of Triatoma infestans in northwestern Argentina. J Med Entomol.

[29] Klotz SA, Shirazi MF, Boesen K, Beatty NL, Dorn PL, Smith S (2016). Kissing bug (Triatoma spp ) intrusion into homes: troublesome bites and domiciliation. Environ Health Insights.

[30] Beatty NL, Klotz SA (2018). The midnight bite A kissing bug nightmare. Am J Med.

[31] Sinelnikov A, Kalinina A, Old JB, Boonlayangoor PW, Reich KA (2013). Evaluation of rapid stain identification (RSID(tm)) reader system for analysis and documentation of RSID(tm) tests. Appl Sci.

